# Deep Learning for Intelligent Assessment of Financial Investment Risk Prediction

**DOI:** 10.1155/2022/3062566

**Published:** 2022-10-11

**Authors:** Yang Sun, Jiajun Li

**Affiliations:** School of Management, Northwestern Polytechnical University, Xi'an 710072, Shaanxi, China

## Abstract

Financial investment promotes the market's fast economic growth and gradually becomes a new trend of social development in the contemporary era. From the national level, financial risk investment activities directly affect the development process of the information technology industry and social and economic benefits. The management of financial risk investment has received more attention, and the types and difficulties of risks are also gradually increasing. Financial regulatory agencies urgently need to establish a sensitive and scientific economic hazard early alarm system. The perfect earlier alarm system stands based on in-depth scientific theoretical research, so studying financial security evaluation and systemic economic earlier alarm systems is of great practical significance. Taking the systemic financial risk as the research object, this paper analyzes the mechanism of financial systemic risk. After that, deep learning technology in financial investment has been used for the first time to reconstruct the index system of financial security evaluation and early warning. The application of deep learning technology in the early warning of systemic financial risks is realized, which provides a reliable basis for the regulatory authorities to build a financial risk early warning system and makes empirical research.

## 1. Introduction

Financial market investment is more challenging, and there are more risk indicators in the financial market. In 2015, China's capital market experienced a significant shock, with the Shanghai Composite Index plummeting by 30%, the Shenzhen Composite Index and the Growth Enterprise Market Index plummeting by 40%, and the market value of the two markets evaporated by more than CNY 20 trillion.

In the context of financial development globalization, financial market transactions between countries are frequent, the financial environment is more complex, and the spread of financial risks is more rapid and extensive. Especially, in the current international financial situation with high leverage, high asset prices, high market volatility, and high risk, financial supervision will become more difficult, and the possibility of a financial crisis outbreak is higher than before. A financial crisis will not only destroy a country's financial system and international financial order but also cause great damage to the real economy, causing an economic crisis, and even cause a serious social and political crisis, endangering national security.

Driven by the new technological revolutions such as big data, cloud computing, meta-universe, and artificial intelligence, machine learning has created a milestone in the development of natural language processing. With the mining process of massive data, financial risk prevention and control is facing the challenge of data sparsity and complexity, and the quantification and prevention of financial risk are very important. When financial institutions become more and more powerful and businesses more comprehensive, accurate quantification and risk reduction of the comprehensive statistical model become more complex than ever. When it is necessary to accurately assess the portfolio risk exposure of large financial institutions, the use of traditional statistical or simulation methods will be increasingly at loss. In order to overcome the shortcomings of traditional methods, researchers began to pay attention to the application of artificial intelligence, machine learning, and deep learning methods in dealing with regional financial risk prevention and control.

The progress of artificial intelligence theory, Internet finance development, and computer technology has brought new financial market forecasting and risk management opportunities. Relevant research work in the theoretical and academic circles has been gradually carried out, and the research methods of financial risks continuously developed are mainly manifested in two aspects. First, from the perspective of machine learning and deep learning of traditional financial risk control, Cavalcante et al. introduced the main research literature since 2009 in 2016 and reviewed the dynamic prediction of financial computational intelligence methods [1]. The full text focuses on financial data preprocessing and clustering, prediction of future market trends, and financial text information mining technology. De Spiegeleer et al. described the machine learning technology of multiple orders of magnitude accelerated by the traditional Gaussian regression, fitted the complex Greek value risk, and summarized the implied volatility, which reduced the calculation times of ordinary option value calculation, American option pricing, and singular option pricing beyond the Black–Scholes model [2]. In 2020, Mashrur et al. introduced a classification method of traditional financial risk management machine learning, detailed its important published literature in the past decade, combed the main confusion faced by researchers, and pointed out the future research direction of traditional financial risk management machine learning [3]. Second, from the perspective of financial system risk control, Helbing emphasizes that today's global network has a wide range of interdependent systems [4]. When the complexity and interaction of the network world increase, these systems may cause serious damage to the society. In 2019, Jones et al. reviewed the existing research methods to measure the risk of the financial system by using ML technology such as big data analysis, network analysis, and sentiment analysis and combed the current research methods and future work directions of risk machine learning in the financial system [5]. In 2020, Zhang et al. described the associated network model of multinetwork structure, focusing on system risk and indirect risk exposure, associating it with direct risk exposure, and capturing and measuring system risk in financial networks [6].

In addition, artificial intelligence is widely used in financial risk control, big data credit, financial market analysis, quantitative investment, and other fields. In terms of the prediction of financial market volatility, Matthew Francis Dixon of the University of Illinois Technology in the United States uses the deep learning neural network to predict the price changes of 43 commodities and foreign exchange futures in the next five minutes. Their information receiver contains 9896 neurons, and there are 135 neurons at the information output end, which are used to deal with the price differences and synergies between different contracts. Ruoxuan Xiong of Stanford University predicted the volatility of the S & P 500 index by integrating Google trends and the market data LSTM model. On the data input, they used the daily S & P 500 index yield and volatility, including 25 Google trends, which included the main areas of the economy. Sirignano of Imperial College London extracted up to 50 TB data from trading data of NASDAQ stock from 2014 to 2015 and established a “spatial neural network” model to predict the quotations of buyers and sellers [6–9]. Some scholars have proposed a two-layer SIR propagation model with an infective medium to analyze the spread of financial shocks. In consideration of strict financial regulation in the A shares, the model assumed that capital cannot flow directly between layers but through the Hong Kong stock market. By applying the model to constituent stocks included in three prominent indices, namely, Standard & Poor 500, Shanghai and Shenzhen 300, and Hang Seng (medium), it established a two-layer Granger networks [10, 11]. These studies show the potential of deep learning financial time series in the presence of a large number of noise data, which makes the application of deep learning in intelligent investment and risk management and control. Quantitative analysis, asset allocation optimization, price fluctuation prediction, market analysis, and other applications are integrated to provide more intelligent investment services.

Financial risk management and control have always been the focus of the financial sector. At present, the most mainstream method in evaluating the financial risks of the financial market is the VaR method, and the prediction of the volatility of the financial market is the most important when using this method [12–16]. The traditional time series analysis is the mainstream method of financial quantitative analysis. The research and application of VaR in China and abroad mainly focus on its valuation method. The valuation method can stand approximately split into three classifications: historical simulation method, analysis method (variance-covariance method), and Monte Carlo (MC simulation method). Financial markets have achieved global integration. Faced with massive financial data, the financial sector needs more accurate market volatility and risk estimates to manage and control the stable development of the financial sector. At the same time, with the advent of the era of big data and the development of Internet finance, the level of financial data has reached a large number of units, and the complexity and unstructured form of data are strengthened. The processing of financial big data is facing great challenges, especially in the processing of complex data such as volatility prediction and risk management. It is difficult for traditional economic models to give specific and accurate results. With sufficient data for learning, deep learning models can often achieve better market volatility forecasting results, significantly reduce human costs, and improve economic hazard control and business processing capabilities. At this time, the fast growth of artificial intelligence, computer, and other technologies provides various possible and practical methods for developing financial markets. The introduction of deep learning into the financial field will bring more intelligent management and production methods. Deep learning will become a new engine for innovation and change in the financial field.

## 2. The Basic Theory of Financial Market

### 2.1. The Basic Theory of Financial Market Investment

Financial market is a large-scale and multilevel complex network, in which many submarkets interact and influence. Finance is the core of economy. Financial market is a market that guides the flow of funds in the economic system and finances funds. Funds are transferred from surplus to shortage departments and fund suppliers and demanders trade in financial markets through credit instruments. Financial market refers to the sum of supply and demand relationship and its mechanism formed by the transaction object of financial assets. Financial markets can be classified from different angles. The most common and important classification is that financial markets are divided into monetary markets and capital markets according to the duration of financial transactions, and both can be subdivided into smaller markets [17–20].

### 2.2. The Basic Theory of Financial Risk

Financial risk refers to a certain amount of financial assets in the future period of expected income loss possibility, which can be divided into market risk, credit risk, liquidity risk, and stock investment risk. In the process of financial operation, financial risks are objective and inevitable. Therefore, the more rapid the development of financial markets, the more prosperous we face financial risks. Financial risks are generally characterized by uncertainty, objectivity, and potential [20–25].

1. The essential feature of financial risk is risk uncertainty, which refers to the negative effects produced in the process of financial activities and the difficulty of fully predicting and mastering various factors in advance
2. Objectivity means that there is a certain necessity for the occurrence of financial risks. Without the will of people to transfer any economic behavior, there is the possibility of latent financial risks.
3. The main reason for the potential financial risks in the financial industry is that there is a serious information asymmetry between financial institutions and investors. Since the specific occurrence is unknown, financial risks have great potential.
4. The financial market we run is actually a network of interconnection, prosperity, and loss. In this network, various financial risks, various financial assets, and institutions are intertwined and closely linked to form a complex system. Various volatility factors will be transmitted between different financial assets; thus, different financial institutions will gradually show similar fluctuations.

### 2.3. Var Risk Measure

Jorion believes that the risk measured by VaR is the maximum possible loss value of assets or portfolios within a certain holding period and at a certain confidence level without abnormal changes in the market. In recent years, VaR has been widely used in the field of risk measurement by research scholars, which is helpful to risk evaluation, risk control, and risk management within financial institutions and has become a common method of risk measurement [26, 27].

VaR is defined as the maximum loss value of assets that may occur at a given confidence level and within a certain holding period. On this basis, in 2008, Adrian proposed the conditional value at risk (CoVaR, Conditional VaR). The method is based on the value at risk, which can be used to measure a financial institution to another financial institution or the whole financial market risk spillover degree. Similar to the value at risk, the conditional value at risk is also at a certain confidence level and a certain period of time. When a single financial institution is at the value at risk, the maximum loss of the entire financial system is at the same. VaR calculation methods are complex and diverse, using three methods, namely, the normal distribution method, the historical simulation method, and the Monte Carlo simulation method.

Different VaR calculation methods have their own advantages and disadvantages. The data obtained by different calculation methods are not the same, and different data results appear. The confidence level of the normal distribution method depends on the subjective judgment of the investigators, which lacks objectivity. The historical simulation method and the Monte Carlo simulation method require a large number of accurate historical data, which requires high calculation ability and accuracy, and the cost is relatively high.

## 3. Financial Investment Market Risk

### 3.1. The Theory of Financial Investment Market Risk Formation

The prosperity and development of the financial industry is accompanied by undertaking, preventing, and resolving financial risks. Financial events may cause adverse financial market fluctuations, loan defaults, financial institutions collapse, fraud, and customer losses. According to the sources of financial risk factors, this paper expands the research context and introduces systemic risk on the basis of traditional financial credit risk, market risk, operational risk, and insurance risk [24, 25]. Financial risk is the potential risk of damage to financial services or functions caused by losses of all or part of financial institutions and financial markets and a serious negative external spillover effect on the economic growth and welfare.

**Figure 1:**
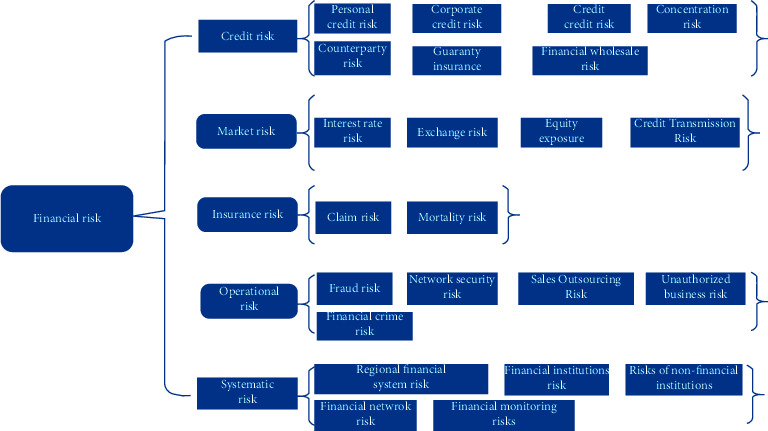
Common financial market risks.

Figure 1 summarizes the types and scope of financial risks from different sources, which helps us to use various regional financial risk prevention and control strategies to measure and resolve the risks of financial complex systems. This new classification perspective helps us focus on regional financial risks and address the risk challenges facing the future financial system.

### 3.2. The Traditional Financial Risk Assessment Method

In order to effectively control and manage the market risks faced by financial institutions, it is necessary to quantify the risks. There are the following common evaluation methods:

1. The simple algorithm (deviation rate and spread rate): The spread rate is a measure of a single securities volatility and risk indicator, and the value of the spread rate is proportional to the size of the stock risk.
2. Sensitivity analysis: There are many market risk factors that affect returns, such as interest rates and exchange rates. The sensitivity in risk measurement measures the sensitivity of the variance of returns to the variance of random variables that affect returns, namely, the ratio of the two.
3. Volatility analysis: The yield is treated as a random variable, and the common methods are variance and standard deviation.
4. The delta-normal division method: The method assumes that the return rate is normal distribution, so the standard deviation of the return rate multiplied by the quantile under the corresponding confidence is equal to the VaR value calculated by the confidence and the corresponding quantile.
5. The historical simulation method: Based on the empirical distribution, this method reconstructs the time series of asset return by using the historical data of financial assets, simulates the future profit and loss distribution of portfolio assets, replaces the real distribution with the historical distribution of return, and calculates the VaR value under given confidence by quantile.
6. The Monte Carlo simulation method: In financial trial production, the Monte Carlo simulation method is the most effective method to calculate the VaR value of financial assets. Regardless of the distribution of financial assets and the nonlinear situation, the results calculated by the Monte Carlo simulation method are satisfactory.

At present, for all kinds of risks in the financial market, improving the existing financial risk measurement methods, accurately fitting the distribution of various financial risks, and discussing the correlation of various financial risks are the focus of many scholars. But it is still not combined with artificial intelligence, deep learning, machine learning, and other new methods. The produced financial investment risk prediction and evaluation method is based on deep learning.

### 3.3. Main Problems in China's Financial Investment Market

At present, China's venture capital industry is in preliminary development. The scale of venture capital cannot meet the needs of the market, and there is no way to fully support the high-tech industry. There are also many difficult problems to be solved in the venture capital industry, which seriously hinders the development of high-tech.

1. In reality, the current venture capital funds cannot meet the market's needs, and the source of funds mainly depends on the national financial allocation and bank technology R & D loans.
2. The circulation of investment capital is the fundamental development model of venture capital, and the circulation of venture capital means that the investment capital has access. Since there is no reasonable and legal capital exit method, venture capital does not achieve the purpose of capital appreciation and a virtuous cycle.
3. The single industrial organization will lead to the accumulation of systemic financial risks. There may be resource depletion problems in the primary industry as the main body, and the lack of timely adjustment of industrial relations may lead to the convergence of industrial relations, which further leads to the occurrence of systemic risks. Secondly, a single industrial structure means that the investment structure is single, and the ability of the industry to resist systemic risks is poor. If there are problems in a certain link in the process of industrial communication, it may lead to a comprehensive risk crisis.

### 3.4. Application of Deep Learning in the Financial Investment Market

With the rapid spread of social media and real-time news media, information retrieval based on instant text is also applied to financial model development. Much useful information can be obtained by analyzing the context of news, financial statements, and corporate information disclosure; so financial text mining research has become very popular in recent years. For example, based on a new event type pattern classification algorithm, different event types of Chinese enterprises are classified. In addition, other input factors are also used to predict the stock price. Based on financial news and stock market data, the LSTM model with transfer learning is realized by using text mining technology. Stock2Vec and TGRU models are also used to generate input data from financial news and stock prices for stock price classification.

The progress of text mining technology in behavioral finance provides the possibility of successfully extracting emotions through online media or social media. People are more and more interested in financial sentiment analysis, especially the sentiment analysis model based on deep learning applied to financial market forecasting. The price of index data and sentiment data in text posts to predict the opening price of stocks the next day can be used. Twitter sentiment data and stock price data to predict the stock prices of Google, Microsoft, and Apple can be used. The sentiment analysis model based on the CNN is used to construct investor sentiment characteristics, and the LSTM model is used to predict a stock trend. A combination of Word2vec and BiLSTM for sentiment analysis and stock timing is considered.

## 4. Financial Market Exchange Rate Forecasting Based on Deep Learning

In the era of big data, there is a trend of integration of big data and the financial industry. At the same time, the emergence of Internet finance not only makes the financial market more complex but also brings massive financial data. When dealing with massive financial data, traditional methods are no longer effective, and big data are the basis for the development of artificial intelligence, so the methods related to artificial intelligence can be introduced into the supervision of financial markets. Deep learning is a method of learning the law of massive data through a deep neural network model, which is known as “the palm of artificial intelligence.” It has been widely used in the financial industry, such as banking handwritten digital image recognition, intelligent customer service, and intelligent investment. Different from the traditional feedforward neural network, deep learning models such as the recurrent neural network (RNN) and the long-short memory neural network (LSTM) not only have strong nonlinear mapping ability but also can realize any complex causal relationship. They also have the memory and can capture the correlation between the time periods before and after the sequence. They are a powerful nonlinear model for processing time series data.

### 4.1. The Financial Market Deep Learning Model

**Figure 2:**
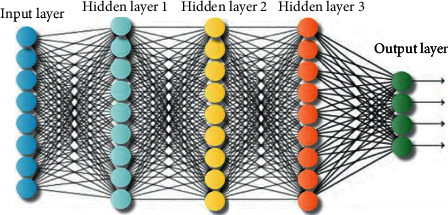
The deep neural network.

Deep learning is considered as a deeper machine learning method, which is composed of the output layer, input layer, and a series of stacked hidden layers between the two layers of the multilayer neural network, as shown in Figure 2. The deep learning method is suitable for solving complex problems, and has been widely used in artificial intelligence tasks in recent years. Compared with general machine learning algorithms, deep learning can extract high-level abstract and complex features through layer-by-layer learning, which can be used as a manifestation of data to improve the accuracy of classification or prediction. For neural networks, the more the data is, the better the training effect of the network, and the more it can reflect the reality. But in practice, it is difficult to select a large number of sample values for training due to the limitation of conditions, and the number of samples is small.

Common deep learning algorithm models include the deep neural network, stacked autoencoders, the deep belief network, the back propagation neural network, and the recurrent neural network.

### 4.2. Data Processing and Model Building

**Figure 3:**
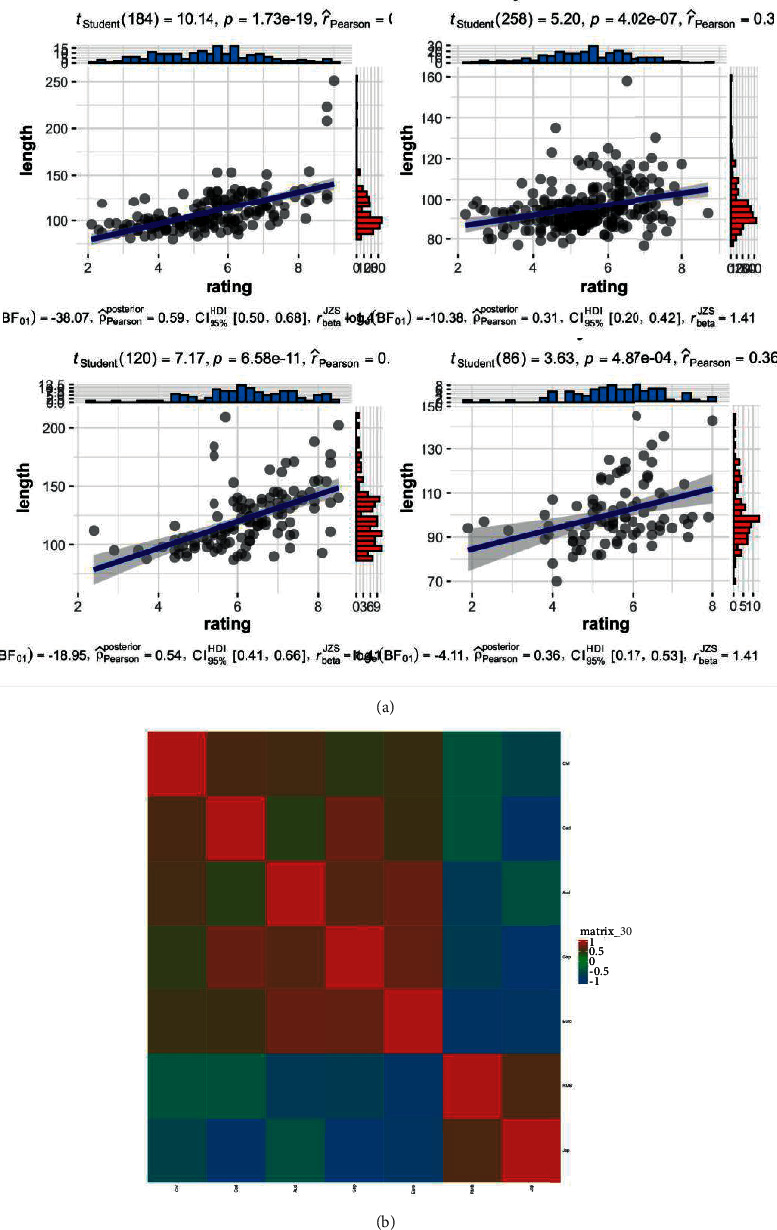
Analysis of some sample characteristics. (a) Partial data distribution and correlation analysis. (b) Heat map of exchange rate correlation matrix of 7 countries.

In this study, before using the deep learning model, we first need to preprocess the data. The study selects the recorded data of seven primary international interaction velocity needs to study the exchange rate prediction ability of the deep learning model in the foreign exchange market. The characteristics of some data are analyzed, as shown in Figure 3. The seven international exchange rate markets are RMB, Euro, Jap, Cad, Aud, Gbp, and Chf, respectively. Each market includes 2409 observations as experimental data.

### 4.3. Exchange Rate Forecasting Based on Deep Learning

(1) The exchange rate prediction of a deep belief network, because the sample is directly used for the test set data, is prone to over-fitting. This paper considers the combination of two models to establish the model.
  (1) $$\begin{array}{c} Q_{t}=\frac{\alpha_{t}+\beta_{t}}{2}\text{,} \end{array}$$
Where *Q*_*t*_ represents the final predicted value, *α*_*t*_ represents the predicted value of ARMA model output, and *β*_*t*_ represents the predicted value of DBN model output.
(2) In this study, the influence of nonlinear characteristics and linear characteristics in historical data is the same, so the prediction results under the integration method are defined as follows:
  (2) $$\begin{array}{c} Q_{t}=\frac{\alpha_{t}+\delta_{t}}{2}\text{,} \end{array}$$
Where *Q*_*t*_ represents the final predicted value, *α*_*t*_ represents the predicted value of ARMA model output, and *δ*_*t*_ represents the predicted value of LSTM model output.

### 4.4. Authentic Proof Analysis

In this study, the TensorFlow framework proposed by Google is used to realize the analysis process of exchange swiftness prediction in the foreign trade market by the deep learning model. By comparing the prediction results of different international exchange rate markets, the structure of the deep learning model with the best prediction performance is determined. Descriptive statistics such as mean, standard deviation, skewness and kurtosis, and the Jarque–Bera normality test and BDS test were used to analyze the historical data of seven international mainstream exchange rate markets.

The following conclusions are obtained by Table 1. In general, the historical data time series of exchange rate forecasting has the characteristics of nonnormal distribution because of the complex linear and nonlinear characteristics. The Jarque–Bera test shows that the historical data time series is nonnormal distribution, while the BDS test shows that the historical data time series are not independent distribution. According to the mean and standard deviation of the first-order difference of the exchange rate, the historical data of the Australian dollar market vary greatly, and the data are scattered. The historical data of the Canadian dollar market, the Swiss franc market, the euro market, the pound market, and the yen market are more concentrated and less discrete. In the seven exchange rate markets, the standard deviation of historical data of the RMB market is the smallest, indicating that the exchange rate change in the market is small and the data are the most concentrated.

**Figure 4:**
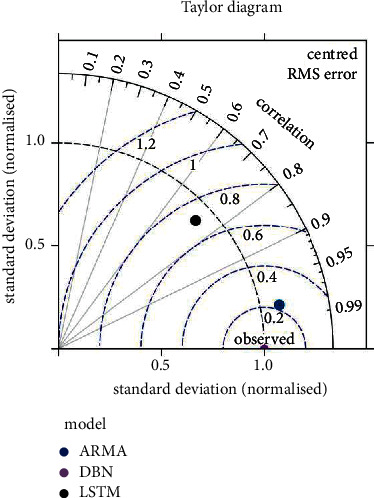
The Taylor model.

Based on Table 2, the following conclusions are drawn. The exchange swiftness forecast outcomes of the DBN model and the LSTM model in the foreign exchange market of seven countries are similar. However, when we divide the international exchange rate into different regions, the application of the model will also change. For example, the LSTM model is more applicable in the Canadian dollar market and the euro market. The DBN model is more applicable in the euro market, the pound market, and the RMB real position. In order to show the advantages and disadvantages of each model more intuitively, The Taylor model is used to evaluate the prediction accuracy of each model for the RMB exchange rate market, as shown in Figure 4.

Overall, the prediction version of the DBN model is better than the LSTM model in the seven exchange rate markets, and the DBN model is more suitable for predicting the exchange rate in the foreign exchange market.

## 5. Establishment of the Integrated VaR Risk Prediction Model Based on Deep Learning

### 5.1. Data Processing

The empirical mode decomposition method is used for data preprocessing.

Step 1: The number of local extremum points and zero crossing pinpoints of the line must be equal or the number difference must be 1
Step 2: At any point in time, the average of the envelopes of the local maximum (upper envelope) and the local minimum (lower envelope) of the IMF must be zero
Step 3: Find all the extreme points of the original data *X* (*t*)
Step 4: The envelope of maximum value (upper envelope) and local minimum value (lower envelope) of original data *X* (*t*) are fitted by cubic spline function)
  (3) $$\begin{array}{c} m\left(t\right)=\frac{m_{\max}+m_{\min}}{2}\text{,} \end{array}$$
Where *m*_max_ represents the upper envelope, *m*_min_ represents the lower envelope, and *m* (*t*) represents the average of the upper and lower envelopes.
Step 5: Subtract the average *m* (*t*) of the upper and lower envelopes from the original data *X* (*t*)
  (4) $$\begin{array}{c} X\left(t\right)-m\left(t\right)=h\left(t\right)\text{,} \end{array}$$
Where *h* (*t*) represents the difference between the original data *X* (*t*) and the average *m* (*t*) of the upper and lower envelopes.
Step 6: To determine whether *h* (*t*) meets the IMF conditions and, if so, to mark *h* (t) as the *i*^th^ lMF, *X* (*t*) is replaced by residual sequence *r* (*t*) = *X* (*t*) − *h*. If not, replace *X* (*t*) with *h* (*t*).
The original data *X* (*t*) can be represented by the sum of all IMFs and the remaining sequence *r* (*t*) at this time
  (5) $$\begin{array}{c} X\left(t\right)=\overset{n}{\underset{i=1}{\sum }}c_{i}\left(t\right)+r_{n}\left(t\right)\text{,} \end{array}$$
Where *r*_*n*_ (t) represents the average or average trend of the original data.
Step 7: *r*_*t*_ = ln (*P*_*t*_/P_*t*−1_) is the daily return rate series, where *P*_*t*_ and *r*_*t*_ represent the daily closing price and daily return rate in period *t*, respectively. The EMD algorithm is used to decompose the time return rate series into several independent IMF, denoted as *r*_*t*_.

### 5.2. Model Establishment

**Figure 5:**
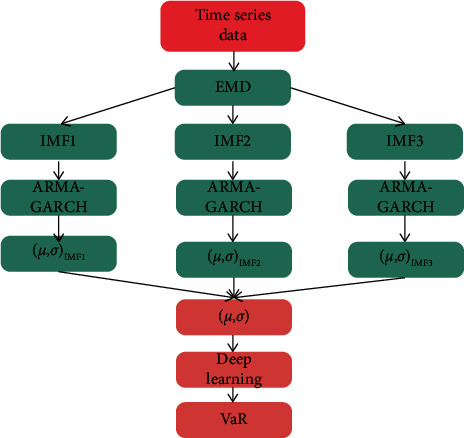
VaR value calculation process.

(1) The mean dependent matrix and the dependent expected deviation matrix of each IMF function and residual sequence at various time scales are estimated. Assuming that the mean dependent value obeys the multiscale ARMA process and *x*_1_, *x*_2_,…, *x*_*n*_ is the observed value of stationary sequence {*X*}, then the mean value *μ* of the ARMA standard is described as follows:
  (6) $$\begin{array}{c} \mu ={EX}_{t},\\ \tilde{\mu }=\frac{1}{N}\overset{N}{\underset{k=1}{\sum }}x_{k}\text{.} \end{array}$$
(2) The integration of conditional mean and conditional standard deviation. According to the accepted values of IMF dependent mean and dependent standard variation under various scales, the nonlinear integration algorithm founded on the deep learning model estimates the total dependent mean and dependent standard deviation.
  (7) $$\begin{array}{c} {\hat{y}}_{t}=\overset{N}{\underset{i=1}{\sum }}\omega_{t}x_{i,t}\text{,} \end{array}$$
Where *y*_*t*_ represents the output reconstruction value at time *t*, *ω*_*t*_ represents the weight of the predicted values of different models, and *N* represents the number of predicted values.
(3) Assuming that the holding period is one day, the time series of the exchange rate market obeys the mean value of *μ* and the variance is *σ*, then the total VaR value in period *t* can be defined as follows: (The flowchart of the VaR calculation is shown in Figure 5).
  (8) $$\begin{array}{c} {VaR}_{t}=\mu_{t}+\sigma_{t}G^{-1}\text{,} \end{array}$$
Where *G*^−1^ represents the reciprocal of cumulative normal distribution.

### 5.3. Prediction Effect Evaluation of the Model

The following conclusions can be drawn from Table 3 data. On the whole, the risk prediction performance of the MLP model is close to that of the DBN model, and both of them perform well in four international exchange rate markets, indicating that the risk prediction performance of these two models is higher than that of the LSTM model which performs well only in two international exchange rate markets. However, compared with the MLP model and the DBN model, the overall VaR prediction of the DBN model exceeds less days, indicating that the performance of the DBN model in risk prediction is higher than that of the MLP model, that is, the DBN model is more suitable for risk prediction than the other two models.

Table 4 is the *P* value result of VaR risk measurement in seven foreign exchange markets.

**Figure 6:**
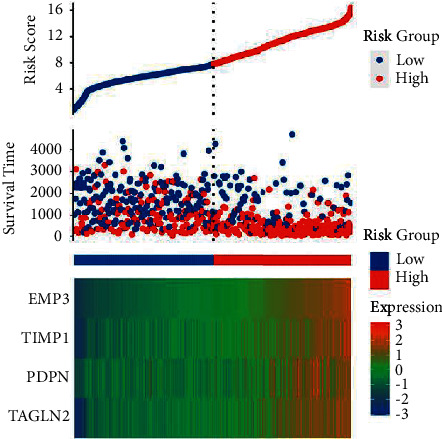
The RMB foreign exchange risk factor.

Based on the *P* value results of VaR risk measurement in seven foreign exchange markets, this paper divides the foreign exchange market in financial investment market into high risk and low risk according to the *P* value results of RMB risk measurement, and the risk factor score is shown in Figure 6.

To sum up, we construct a foreign exchange market risk measurement model based on deep learning. After the EMD method is used to decompose the historical data in different time scales, the ensemble algorithm is combined with the ARMA-GARCH model and the deep learning model. The ARMA-GARCH model is used to calculate the conditional mean and conditional standard deviation under different time scales. Then, the ensemble algorithm is used to calculate the total conditional mean and conditional standard deviation. Finally, the deep learning model is used to calculate the overall VaR value. Because the risk measurement method proposed in this chapter calculates the risk of IMF at different time scales in different international exchange rate markets, the measurement of financial investment risk is more accurate.

## 6. Conclusion

Financial risk investment is very important for investors. Events with high risk of capital investment will cause irreparable losses. How to achieve stable financial investment is a major problem. This paper proposes a prediction and evaluation method of financial market investment analysis based on deep learning. In exchange rate forecasting, this study used historical data from seven international mainstream exchange rate markets, including the RMB, the euro, the yen, the Canadian dollar, the Australian dollar, the pound, and the Swiss franc. In the training stage, the training set data is used to train the deep belief network model and the long-term and short-term memory model in the recurrent neural network model so as to determine the parameters of the prediction model, such as the number of hidden layers, the number of nodes, and the learning rate and construct the deep learning network structure with better prediction performance. In the prediction stage, the test set is used as input data to make the deep learning model predict the test set, and the results are compared with the reference model, the ARMA model, so as to judge the prediction performance of the model. The empirical results show that the performance of a deep belief network in exchange rate prediction is better than that of a circular neural network.

In terms of VaR risk measurement, this study applies seven international mainstream exchange rate markets and calculates VaR risk measurement values by applying the deep belief network model, the deep multilayer perceptron model, and the recurrent neural network model. The risk measurement value shows that RMB is currently in a low-risk range. Finally, the advantages of the VaR risk measurement model of the deep confidence network model are verified, and a reference model is made for the exchange rate risk assessment of the financial investment market.

## Figures and Tables

**Table 1:** Descriptive statistical analysis of 7 foreign exchange markets.

	Aud	Cad	Chf	Euro	Gbp	Jap	RMB
Mean value	2.99	2.92	−17.88	−0.48	−12.79	−9.48	−11.69
Variance	0.01	0.007	0.007	0.006	0.006	0.007	0.001
Deviation	−0.67	−0.06	0.76	0.21	−0.37	−0.28	0.01
Peak	14.35	8.54	16.44	5.98	9.08	7.71	117.41
JB test	0.001	0.001	0.001	0.001	0.001	0.001	0.001
BDS test	—	—	—	—	—	—	—

**Table 2:** Foreign exchange market exchange rate forecast results.

Model	Aud	Cad	Chf	Euro	Gbp	Jap	RMB
ARMA	4.9356	3.1017	6.3702	3.6471	4.2829	4.3479	0.34035
DBN	4.9391	3.1009	6.2669	4.2764	4.2661	4.3490	0.33356
LSTM	4.9897	3.0836	6.3020	3.6844	4.9176	4.4498	0.34703

**Table 3:** VaR risk measurement results of 7 foreign exchange markets.

VaR measuring model	Aud	Cad	Chf	Euro	Gbp	Jap	RMB
ARMA-GARCH	26.67	17.00	12.00	22.23	29.67	28.33	23.00
MLP	5.33	8.00	2.00	4.33	19.00	16.33	5.67
DBN	6.00	8.33	0.67	4.33	21.00	1633	4.67
LSTM	8.00	6.00	5.33	5.00	23.33	16.67	4.67

**Table 4:** *P* value results of VaR risk measurement in 7 foreign exchange markets.

VaR measuring model	Aud	Cad	Chf	Euro	Gbp	Jap	RMB
ARMA-GARCH	0.0000	0.0341	0.0485	0.0411	0.0001	0.0023	0.3451
MLP	0.5952	0.3005	0.0318	0.4939	0.0000	0.0008	0.5856
DBN	0.6517	0.4177	0.0126	0.4939	0.0000	0.0018	0.4343
LSTM	0.4347	0.5178	0.5952	0.3242	0.0000	0.0017	0.4334

## Data Availability

The data used to support the findings of this study are available from the corresponding author upon request.
